# Screw Analysis, Modeling and Experiment on the Mechanics of Tibia Orthopedic with the Ilizarov External Fixator

**DOI:** 10.3390/mi13060932

**Published:** 2022-06-11

**Authors:** Peng Su, Sikai Wang, Yuliang Lai, Qinran Zhang, Leiyu Zhang

**Affiliations:** 1 School of Electromechanical Engineering, Beijing Information Science and Technology University, Beijing 100124, China; supeng@bistu.edu.cn (P.S.); skywang0306@foxmail.com (S.W.); laiyuliang666@163.com (Y.L.); zhangqinran@bistu.edu.cn (Q.Z.); 2 Beijing Key Laboratory of Advanced Manufacturing Technology, Beijing University of Technology, Beijing 100124, China

**Keywords:** Ilizarov external fixator, screw theory, orthopedic treatment, tension of broken bone, stress distribution

## Abstract

The Ilizarov external fixator plays an important role in the correction of complex malformed limbs. Our purpose in this work was to reveal the transmission of adjustable forces between the external fixator and the broken bone, and express the stress distribution at the end of the broken bone during the orthopedic treatment. Firstly, the screw model of the fixator was established and the theoretical relationship between the adjustable force and the stress was obtained. A sheep tibia was taken as a representative research object and its ediTable 3D entity was obtained by CT scanning. Then the mechanical model of the fixator and tibia was built using the ABAQUS software. Correction experiments were performed on the sheep tibia to measure the adjustable/support forces and tensions of the tibia. The measured results were imported to the screw and mechanical model, and the theoretical and simulation values were calculated. The theoretical tensions calculated by the screw model had a similar shape and doubled the value compared with that of the measured results. The transfer efficiency between the two results was improved and kept at about 50% after the initial 2~3 periods. The maximum stress occurring at the surface of the broken bone end was near the Kirschner wire pinhole. The simulation results for the tensions from the mechanical model showed a similar change trend, and the value was slightly higher. A biomechanical model of the Ilizarov external fixator was derived and verified through calculations, simulations and experiments. The change law of the adjustable forces and the tensions existing in the broken sheep tibias is presented herein, and offers a helpful contribution to orthopedic treatment.

## 1. Introduction

As a surgical instrument, the Ilizarov external fixator plays an important role in the correction of complex malformed limbs. It can adjust the configuration of the bone external fixator according to the treatment progress, and may affect the results of surgery in the orthopedic process. Watson proposed that the engineering structure of the external fixator is related to bone healing [1]. However, this orthopedic treatment is mostly based on doctors’ experience, and its orthopedic mechanism, including the tension at the broken bone and the transfer efficiency, has not been well studied. A mechanical study of the external fixator can reveal the mechanical relationship between the adjustable force and the broken bone end, which is helpful for doctors in adjusting external fixators [2].

As a practical and simple theoretical calculation method, screw theory has attracted the attention of many researchers and has been widely used in kinematics and static analysis of the mechanism. Based on the screw theory, Niu J, Wang H, Shi H, et al. analyzed the constraints and basic motion of the leg mechanism and solved the kinematics of the leg mechanism [3]. Researchers from Beihang university proposed an analytical algorithm for measuring three-dimensional forces and three-dimensional torques, which can achieve high precision measurement of interactive forces on the Stewart platform [4]. Cunfeng Kang and Zixiao Liu et al. proposed a position transformation method that can generate circular trajectory more accurately [5]. To investigate the motion around the libration points, Yingjing Qian, Zixiao Liu et al. transformed the origin of the coordinate system from the center of the system to translational points through coordinate transformation [6]. Further study of screw theory reveals that it also provides various feasible schemes in the configuration of parallel mechanism, such as 3CRU translational parallel mechanism, 2-UR-RRU parallel robot, etc. [7,8], which provide ideas for theoretical analysis and experimental study of the Ilizarov external fixator.

Due to the special environment of bone injury in the body, it is impossible to reproduce the true intervention process of orthopedic devices on broken bones. Therefore, many researchers have studied the relationship between various orthopedic devices and fracture healing through finite element analysis. The axial tension and compression of the tibia are real-time detected by simulating the actual motion state of the bone external fixator [9]. Karunratanakul K used a finite element model to predict the stiffness of the fixer [10]. Malayan researchers used polyethylene tubes to represent the tibia bone and applied the above methods to study the mechanical behaviors of different configurations of bone external fixators [11]. Although the various configurations and biological environment of the fixator have been discussed above, few of them have studied the stress and strain of the fixator and the tibia’s broken end during the entire orthopedic process.

The mechanical model of the Ilizarov external fixator is the basis of numerical simulation, which can provide better prediction of mechanical properties and comparative analysis of the correction process. This study intended to establish an accurate mechanical model of the fixator and simulate the force transmission process in the normal fracture healing process using the screw theory. A tibia fracture correction and recovery experiment was performed on a special experimental bench. Referring to Cunfeng Kang and Zixiao Liu et al., the finite element analysis of the welding pipeline was carried out and compared with the experiment of [12], the measured data were imported into the mechanical model and the loading process was simulated using ABAQUS software. The simulated and actual tensions and stresses of the tibia were compared to improve understanding of the force transmission relationship between the Ilizarov external fixator and the broken tibia end.

## 2. Materials and Methods

### 2.1. Screw Model of Ilizarov External Fixator

The Ilizarov external fixator has various configurations in clinical applications. The specifications of the hole rings and the diameters and angles of the Kirschner wires effect the healing of broken bone tissue [9,13] and are determined according to the actual situations of patients. The corresponding classic parameters are shown in Table 1.

In orthopedic treatment, the mechanical analysis between the adjustable rods and the tibial broken end is insufficient, and it is difficult to achieve quantitative orthopedic and precise assessment, which greatly hinders the corresponding applications. The general kinematics analysis method is the D-H parameter method, but this method has a fundamental defect: all the motion is in the *x*-axis and *z*-axis, while it cannot represent the motion in the *y* axis. Therefore, it is necessary to use another method to establish the kinematics model: the screw theory. Using the screw to describe the kinematics of the rigid body has two advantages: first, it only needs two coordinate systems—the basic coordinate system and the tool coordinate system—to describe the motion of the rigid body from the whole, so as to avoid the singularity caused by using a local coordinate system in the D-H parameter method; secondly, the screw method describes the geometric meaning of rigid body motion clearly, which avoids the disadvantages of abstract mathematical symbols, thus greatly simplifying the analysis of the mechanism [14]. The screw theory can describe the rigid motion of each joint as a rotation around the joint axis and a translational motion along this axis. In the existing external fixator, only two of the screw coordinate systems of the broken bone end and the adjustable/support rod with respect to the base platform need to be established to avoid the singularities of the traditional D-H parametric method and simplify the kinematics and dynamics analyses [15]. Meanwhile, the rigid motion in the orthopedic treatment can be clearly described.

In the kinematics analysis, the motion screw and wrench represent an instantaneous motion of a rigid body and the force/moment acting on a rigid body, respectively, where there exists a dual relationship between them [16,17]. Hence, a set of dual vectors in space can represent the angular/linear velocities in the kinematics and the force/moment in rigid-body mechanics. The wrench can be compounded by a force along an axis and a torque around the axis. The generalized force acting on the rigid body consists of a moving component *f* (pure force) and a rotating component *τ* (pure torque), which can be expressed in a six-dimensional vector in the inertial coordinate system [18]:

(1) $$F=\left(\begin{array}{c} f\\ \tau \end{array}\right)=\left(\begin{array}{c} s\\ r\times s+hs \end{array}\right),\;\;\;h\;is\;finite,$$

(2) $$F=\left(\begin{array}{c} 0\\ \tau \end{array}\right)=\left(\begin{array}{c} 0\\ s \end{array}\right),\;h=\infty ,$$

where *f*, *τ*∈*R*^3^, h is called the pitch and denotes the ratio of moment to force, *r* × *s* is the distance of the axis of rotation from the origin of the inertial coordinate system {*o*_1_}, and *F* denotes the wrench along the screw motion *s*.

**Figure 1:**
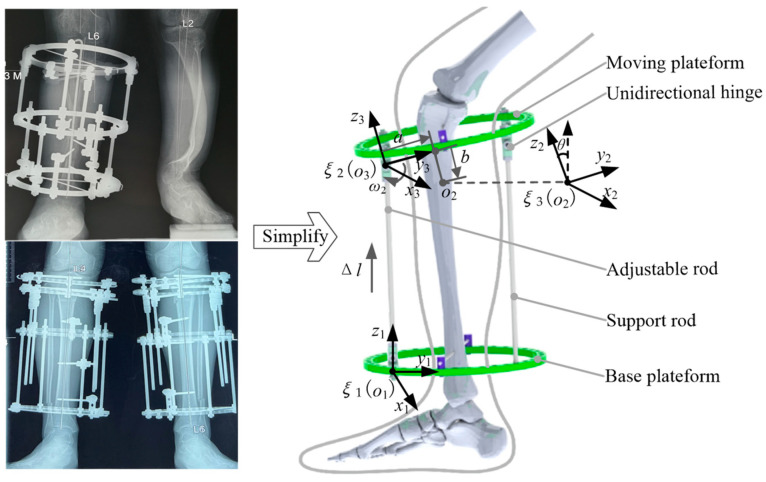
Kinematic model of Ilizarov external fixator.

The adjustable and support rods are defined as two kinematic chains by the screw theory. Since the adjustable and support rods are symmetrical and parallel to each other, they possess the same kinematic features, and only one of them needs to be analyzed in detail. During the orthopedic treatment, the movements of the kinematic chain can be decomposed into a translation motion along the rod and a rotational motion around the hinge. The adjustable forces can be transmitted to the moving platform and achieve predetermined orthopedic movements of the severed tibia. The inertial coordinate system {*o*_1_} of the adjustable rod and the coordinate system {*o*_2_} of the broken tibia end are established on the initial posture of this fixator, as shown in Figure 1.

During the orthopedic period, the adjustable rod only has a translational motion along the axis *o*_1_*z*_1_, and the spinor *ξ*_1_ of the rod can be expressed as:

$$w_{1}=\left(\begin{array}{c} 0\\ 0\\ 0 \end{array}\right),v_{1}=\left(\begin{array}{c} 0\\ 0\\ 1 \end{array}\right),\xi_{1}=\left(\begin{array}{c} 0\\ v_{1} \end{array}\right)=\left(\begin{array}{c} 0\\ 0\\ 0\\ 0\\ 0\\ 1 \end{array}\right),$$

where *w*_1_ and *v*_1_ are defined as the angular/linear velocity of the adjustable rod. However, the adjustable rod moves only along the axis *o*_1_*z*_1_ in the initial stage of orthosis. The spinor *ξ*_1_ only contains the velocity *v*_1_, which is straight up, and the angular velocity *w*_1_ is equal to zero.

The upper end of the adjustable rod is hinged to a unidirectional hinge. The unidirectional hinge has a rotational motion around the axis *o*_2_*x*_2_, where the corresponding spinor *ξ*_2_ is:

$$w_{2}=\left(\begin{array}{c} 1\\ 0\\ 0 \end{array}\right),r_{2}=\left(\begin{array}{c} 0\\ 0\\ l \end{array}\right),\xi_{2}=\left(\begin{array}{c} w_{2}\\ r_{2}\times w_{2} \end{array}\right)=\left(\begin{array}{c} 1\\ 0\\ 0\\ 0\\ l\\ 0 \end{array}\right),$$

where *w*_2_ and *v*_2_ are defined as the angular/linear velocity of the unidirectional hinge, and *r*_2_ denotes the corresponding position in the coordinate system {*o*_1_}.

The Lie algebra ${\hat{\xi }}_{i}$ (*i* = 1, 2) attitude change matrix $e^{\theta_{i}{\hat{\xi }}_{i}}$ of the spinor $\xi_{i}$ can be calculated:

(3) $${\hat{\xi }}_{i}=\left[\begin{array}{cc} {\hat{\omega }}_{i} & v_{i}\\ 0 & 0 \end{array}\right],$$

(4) $$e^{\theta i\hat{\xi }i}=\left[\begin{array}{cc} e^{\theta_{i}{\hat{w}}_{i}} & \left(I-e^{\theta_{i}{\hat{w}}_{i}}\right)\left(w_{i}\times v_{i}\right)+\theta_{i}\cdot w_{i}\cdot w_{i}^{{}^{T}}\cdot v_{i}\\ 0 & 1 \end{array}\right],$$

Substituting Equation (4) into Equation (5), the function of $e^{\theta_{i}{\hat{\xi }}_{i}}$ with respect to Δ*l* can be solved:

(5) $$e^{\theta_{1}{\hat{\xi }}_{1}}=\left(\begin{array}{cccc} 1 & 0 & 0 & 0\\ 0 & 1 & 0 & 0\\ 0 & 0 & 1 & \Delta l\\ 0 & 0 & 0 & 1 \end{array}\right),$$

(6) $$e^{\theta_{2}{\hat{\xi }}_{2}}=\left(\begin{array}{cccc} 1 & 0 & 0 & 0\\ 0 & \cos\theta & -\sin\theta & l\sin\theta \\ 0 & \sin\theta & \cos\theta & l\left(1-\cos\theta \right)\\ 0 & 0 & 0 & 1 \end{array}\right),$$

where *θ_i_* denotes the rotation angle of the unidirectional hinge and *l* and Δ*l* are the length and the change of the adjustable rod, respectively.

Substituting Equations (6) and (7) into the exponential product formula, the posture of the fixator can be obtained:

(7) $$\begin{array}{ll} p\left(\theta \right) & =e^{\theta_{1}{\hat{\xi }}_{1}}e^{\theta_{2}{\hat{\xi }}_{2}}p\left(0\right)\\ & =(\begin{array}{cccc} 1 & 0 & 0 & 0\\ 0 & \cos\theta & -\sin\theta & l\sin\theta \\ 0 & \sin\theta & \cos\theta & \Delta l+l\left(1-\cos\theta \right)\\ 0 & 0 & 0 & 1 \end{array}), \end{array}$$

where *p*(*θ*) is the attitude matrix of the unidirectional hinge and *p*(0) is the attitude matrix of the initial posture in the coordinate system {*o*_1_}:

$$p\left(0\right)=\left[\begin{array}{cc} I & \left(\begin{array}{c} 0\\ l\\ 0 \end{array}\right)\\ 0 & 1 \end{array}\right].$$

The upper broken tibia is installed on the moving platform through the Kirschner wires. The moving platform drives the upper broken tibia to achieve the correction function with help of the hinge. The coordinate system {*o*_3_} is established at the center of the hinge. The coordinate system {*o*_2_} of the broken tibia end is obtained through shifting the coordinate system {*o*_3_} by the displacements *a* and *b* along the axes *o*_3_*y*_3_ and *o*_3_*z*_3_. Similarly, according to the transformation formula of the motion screw, the relationship between the wrenches *F*($f_{i}$ $\tau_{i}$) (*i* = 1, 2) and *F*($f_{o_{2}}$ $\tau_{o_{2}}$) of the adjustable rod and the broken tibia end can be established.

(8) $$\begin{array}{ll} F\left(f_{o_{2}}\;\tau_{o_{2}}\right) & =R_{x}F\left(f_{i}\;\tau_{i}\right)\\ & =\left(\begin{array}{ccc} 1 & 0 & 0\\ 0 & \cos\theta & -\sin\theta \\ 0 & \sin\theta & \cos\theta \end{array}\right)F(f_{i}\;\tau_{i}), \end{array}$$

where $f_{i}$ and $\tau_{i}$ denote the force and the wrench in the coordinate system {*o*_1_}.

### 2.2. Mechanical Model of Ilizarov External Fixator

**Figure 2:**
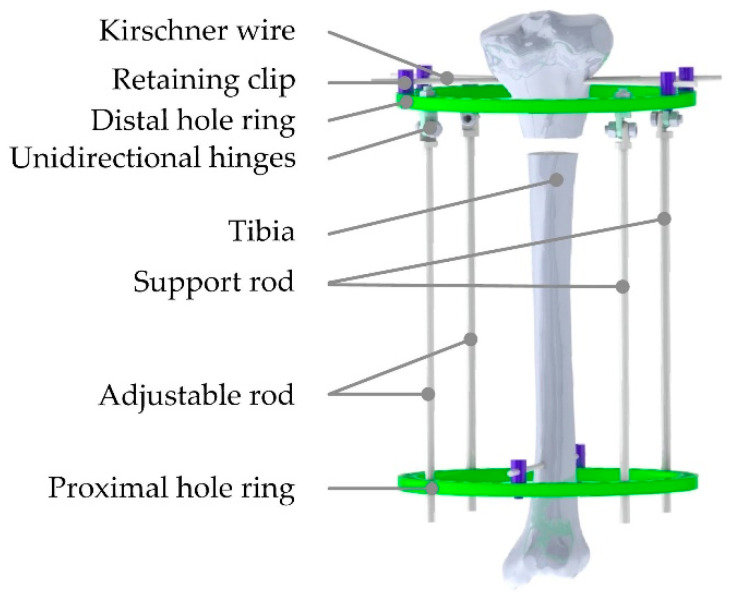
Schematic diagram of simulation model.

A finite element model of the Ilizarov external fixator needs to be established corresponding to the screw model. In order to reduce the error caused by the clearance, the connection part is simplified, and the external fixator is divided into seven main parts: base platform, moving platform, adjustable rod 1, adjustable rod 2 and support rod3, support rod4, and tibia, as shown in Figure 2. At the same time, the operation is simplified and the force is applied directly on the adjustable rod, so that the measured value obtained is more accurate. The proximal and distal hole rings are the base and moving platforms. Two pieces of the broken tibia are fixed by Kirschner wires, which are installed on the hole ring through retaining clips. The adjustable and support rods are inserted in the proximal hole ring and locked during the orthopedic treatment. Similarly, the rods are connected to the distal hole ring through the unidirectional hinges. The hinges are inserted in the distal hole ring and fixed by nuts. The hinges, nuts and distal hole rings are taken as a part, then the rods and the proximal hole ring are taken as another part. The moving platform can rotate around the axes of the two hinges connected to the support rods.

The original 3D model of the sheep tibia is taken as the subject and obtained by CT scanning. The ediTable 3D entity can be generated by the softwares Mimics and Geomagics. The tibia entity is imported into the ABAQUS software to analyze its mechanical properties. The Ilizarov external fixator is made of stainless steel, which possesses the properties of the linear elasticity and isotropy. The tibia is composed of cancellous and compact bones, and osteoporosis affects their function, distribution areas and load capacity [19]. Hence, the tibia can be simplified as an isotropic material composed of cancellous and compact bones. The proper values of the Poisson ratio λ and elastic modulus E are assigned to the tibia model.

CT images can completely and accurately reflect the relationship between material characteristics and gray values. The CT images are imported into Mimics software and the values of the parameters are assigned to the mesh model. There is a linear relationship between the apparent density *ρ* of human bones and the gray value *HU* [20,21]:

(9) $$\rho =\rho_{\max}HU/HU_{\max},$$

where $\rho_{\max}$ and *HU*_max_ are the maximum of the parameters ρ and *HU*. Further, the relationship between the apparent density ρ and the elastic modulus *E_i_* is as follows [22]:

(10) $$E_{1}/\mathrm{MPa}=2065*{\left[\rho /\left(g\cdot cm^{-3}\right)\right]}^{3.09},$$

(11) $$E_{2}/\mathrm{MPa}=1904*{\left[\rho /\left(g\cdot cm^{-3}\right)\right]}^{1.64},$$

where *E*_1_, *E*_2_ are the elastic moduli of compact and cancellous bones. The values of Poisson ratio λ of dense and cancellous bones are equal to 0.3.

**Figure 3:**
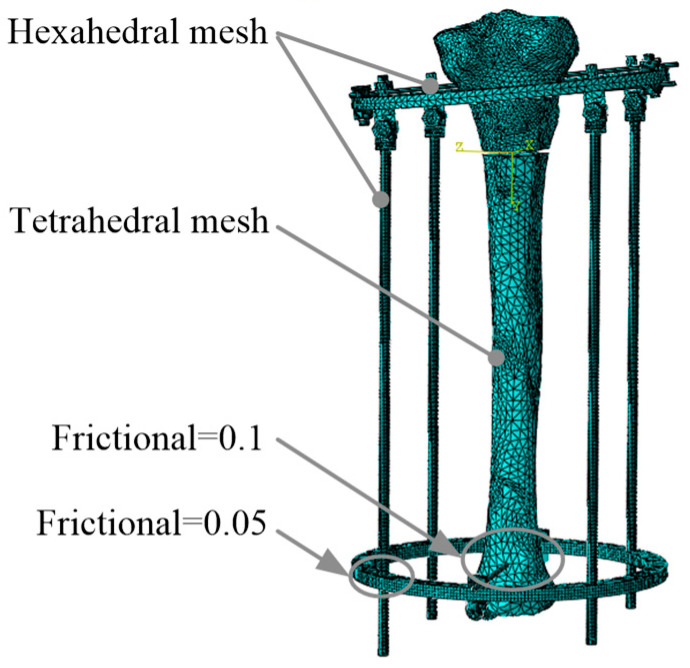
Finite element model of tibia orthopedic.

The binding constraints are adopted in the bolts and nuts of the unidirectional hinges to simulate the behavior of bolt fastening. Finite element model parameters of the Kirschner wire, steel wire holder, and tibia are shown in Table 2, and the finite element model is shown in Figure 3.

The frictional coefficient between stainless steels was defined as 0.05. The tibia and Kirschner wires were defined as the common surface-to-surface contact and the frictional coefficient was equal to 0.1. During the adjustment of the fixator, the base platform was fixed and the osteotomy section was kept stationary relative to the base platform. The base platform and osteotomy section were completely constrained to simulate the fixation of the tibia to the moving platform.

### 2.3. Measurement of Orthopedic Forces

**Figure 4:**
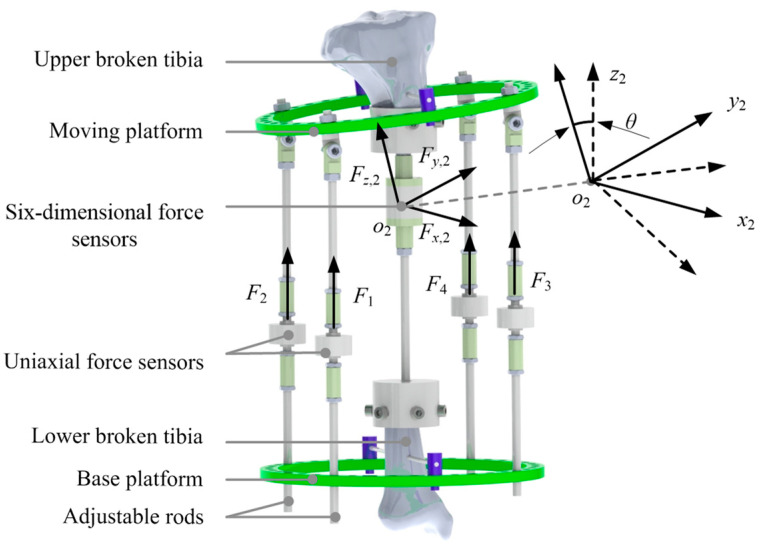
Installation of force sensors.

The sheep tibia was taken as the research object in the orthopedic experiments. Compared with human tibia, the sheep tibia is of smaller diameter and higher hardness, matching the tensile strength of animal experiments [23,24]. Meanwhile, the sheep tibia can withstand greater stress over long periods of time. The tibia was taken from a sheep without obvious anatomical abnormalities that had been slaughtered within 3 h previously. The tibia was at an initial deviation angle of 6.85°, and was cut into two pieces in the osteotomy. The two pieces were installed to the fixator by the Kirschner wires. A six-dimensional force sensor was installed at the osteotomy site to accurately measure the tensions along three directions, as shown in Figure 4. A uniaxial force sensor was serially installed in each rod to measure the adjustable or support force. During the installation of the sensors, the tibia should be kept in the middle position and vertical posture.

The process of fracture healing was simulated by changing the lengths of the two adjustable rods. According to the pulling speed in Table 1, the osteotomy surface of the upper broken tibia and the moving platform were rectified to the position parallel to the base platform by the standard limb lengthening methods [25].

**Figure 5:**
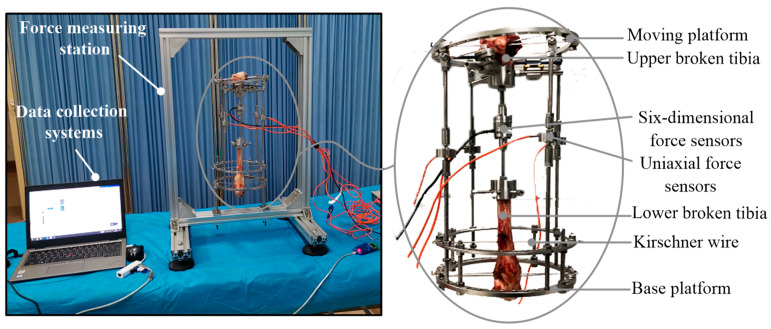
Measurement platform of the sheep tibia.

The adjustable forces *F*_1_ and *F*_2_ were measured by the installed sensors and the support forces *F*_3_ and *F*_4_ were obtained. The tensions $F_{x,2}^{m}$ $F_{y,2}^{m}$, $F_{z,2}^{m}$ between the upper and lower broken tibias along the three axes of {*o*_2_} were measured. The pre-tightening forces of the fixator and the osteotomy site were measured at the beginning of the orthopedic process. During the orthopedic experiments, the adjustable/support forces *F_i_* and the tensions were measured and recorded in real time, as shown in Figure 5. Ten orthopedic experiments were completed and the measurement data had similar variation laws and consistency.

## 3. Results

### 3.1. Orthopedic Force Analysis

The adjustable rods were taken as the actuators to simulate the orthopedic treatment. The adjustable and support forces *F_i_* in the rods had significant effects on the orthopedic treatment of the tibia. The tension $F_{z,2}^{m}$ at the broken tibia is an important factor to stimulate the process of the bone regeneration which should be continuous and periodic. Besides, the frequency of adjusting the rods and magnitude of the tension also should be appropriated [26,27]. Excessive tension will lead to bone disorders in the extension area of the broken bone. Otherwise, the lack of tension will lead to the healing of the broken tibia ahead of schedule [28].

**Figure 6:**
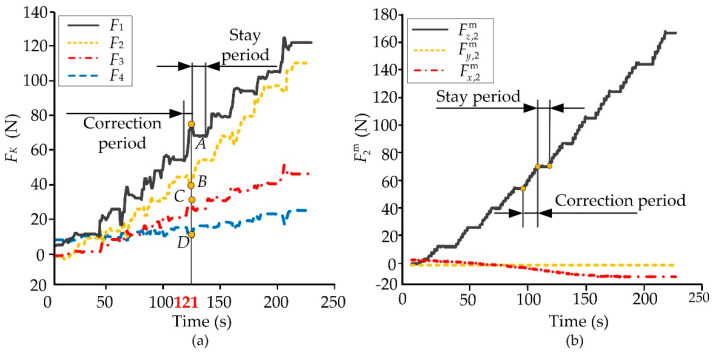
Adjustable/support forces and tensions of the fixator. (**a**) Adjustable and support forces on each rod. (**b**) The measured value of pull tension.

The adjustable and support forces *F_i_* during the orthopedic process are shown in Figure 6. The entire treatment is divided into 10 periods, each lasting one day. A period consists of correction and stay stages. The adjustable nut is rotated a single revolution for each correction stage, and the length of the rod is lengthened or shortened by 1 mm. As patients experience pain when the nut is rotated, a stay stage is added between two correction stages. In the stay stage, the adjustable and support forces remain almost unchanged in order to relieve the pain of patients. The clinical treatment of bone correction lasts 10 days, but it is difficult to work with fresh sheep tibias for such a long time. Hence, each period in the simulated experiment was compressed to 24 s.

The changes in the forces emerging in the rods and tibia are shown in Figure 6. The adjustable rods used as actuators aggravated the generation of internal stress in the fixator. The forces *F*_1_ and *F*_2_ first increased and then decreased to a stable level during the correction stages. The support rods as passive components shared the loads of the fixator. The forces *F*_1_ and *F*_2_ were determined by the load distribution during the whole treatment. Since the load distribution was closely related to the initial pre-tightening state, and there were losses between the adjustable force and the support force, the support forces were always less than the adjustable forces. Meanwhile, during the correction stages, the changing trend of the support forces was opposite to that of the adjustable forces. All the four forces *F_i_* remained unchanged during the stay stage. The sixth correction period was selected, and a dotted line was added at the time of 121 s (Figure 6a). The cross points of the dotted line and the force curves denoted by A, B, C, and D were extreme points of the four curves in this period. It can be seen that the four rods with motion consistency restricted each other. The tensions between the two broken pieces of tibia are depicted in Figure 6b. The tension $F_{z,2}^{m}$ along the axis of the broken end, which was important to the correction area, had similar changes compared with that of the forces *F*_1_ or *F*_2_. The tension $F_{z,2}^{m}$ increased gradually in the correction stage and remained unchanged in the stay stage. The other horizontal components, $F_{x,2}^{m}$ and $F_{y,2}^{m}$, which were harmful to the orthopedic treatment, remained at small values.

The load applied to the fixator through the adjustable rods and support rods was about 300 N, and the average stress transmitted to the broken bone end was 140 N. Compared with Gessmann’s results [29] of indirectly pressurizing the four rings Ilizarov fixator with 300 N and the load on the broken bone end with 110–120 N, the results were slightly different, due to certain differences in structure and pressurization method. However, the similar trend proves the correctness of the experimental data.

### 3.2. Orthopedic Force Theoretical Calculate

**Figure 7:**
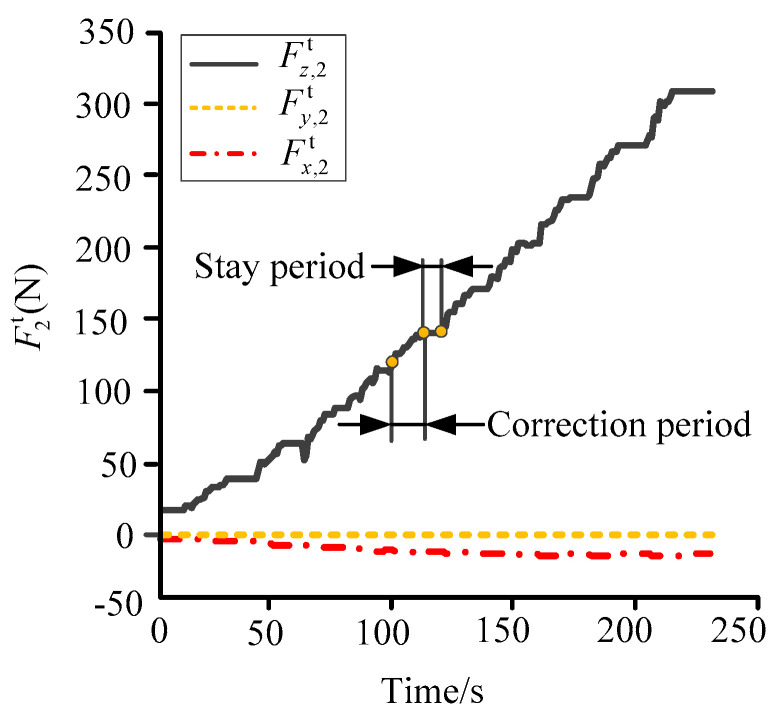
Theoretical tensions at the osteotomy.

The forces *F_i_* and the rotation angle *θ* were substituted into Equation (8) where the rotation angle *θ* varied uniformly from 6.85° to 0°. The theoretical tensions at the extension area of the broken bone were calculated by the software Matlab, as shown in Figure 7. 

The influences of pre-tightening, frictions in the fixator and the motion synchronization of the adjustable rods were disregarded in the theoretical calculation. The theoretical tensions $F_{z,2}^{t}$ at the correction and stay periods were consistent with the measured tension $F_{z,2}^{m}$. In addition, the other two tensions $F_{x,2}^{t}$ and $F_{y,2}^{t}$ all had a similar shape to that of $F_{x,2}^{m}$ and $F_{y,2}^{m}$ in the experiments. Hence, the screw model established in Section 2.1 is suitable for the actual movements of the fixator and has good simulation accuracy. However, the maximum of $F_{z,2}^{t}$ was nearly twice that of $F_{z,2}^{m}$. This was because the influences mentioned above led to the loss of the adjustable and support forces.

The transfer efficiency *η_z_* between the measured and theoretical forces was defined to evaluate the efficiency of converting the adjustable and support forces into the tensions of broken tibia in orthopedic treatment, where *η_z_* could be obtained by:

(12) $$\eta_{z}=\frac{F_{z,2}^{t}}{F_{z,2}^{m}}.$$

**Figure 8:**
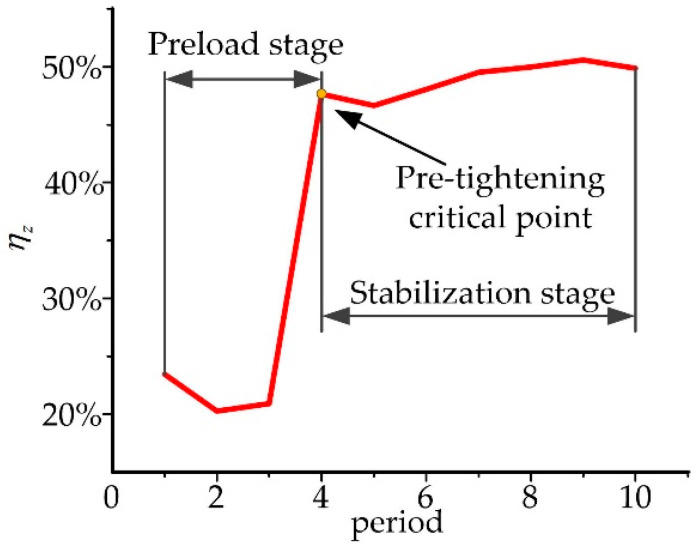
Transfer efficiency *η_z_*.

The index *η*_z_ was calculated based on the obtained data in Figure 6b and Figure 7, as shown in Figure 8. In the first 50 s of the experiment, the adjustable and support forces *F_i_* were used for the pre-tightening of the fixator and rods. Few tensions were transmitted to the extension area of the broken bone. After 2~3 periods, the transfer efficiency *η_z_* was improved and kept at about 50%.

### 3.3. Stress Distribution of Ilizarov External Fixator and Tibia

**Figure 9:**
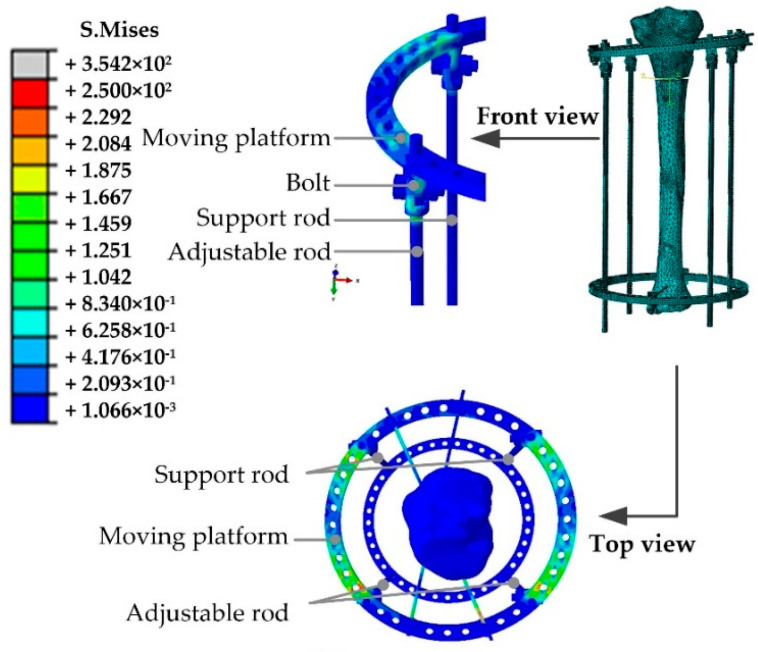
Stress distribution of Ilizarov external fixator at the tenth correction period.

In order to analyze the stress distribution of the fixator and tibia, a finite element model of this mechanical system was established to simulate the correction periods under the software of ABAQUS, as shown in Figure 9. 

**Figure 10:**
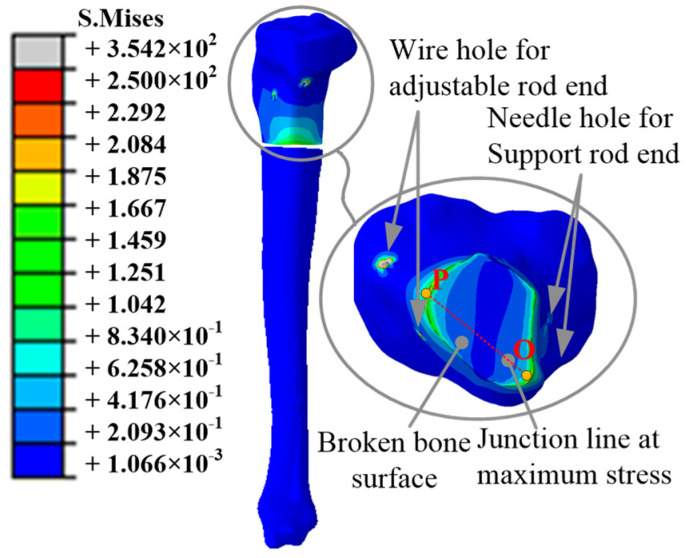
Mises stress state diagram of tibia.

The mean value of the two adjustable forces *F*_1_ and *F*_2_ in each correction period was applied to the end of the adjustable rod, as shown in Table 3. The tension $F_{z,2}^{s}$ in the tenth correction period reached the peak value, which had the most obvious stretch effect on the tibia end. Hence, this correction period was selected for further analysis, as shown in Figure 10. The adjustable forces were transferred from the adjustable rods to the Kirschner wires, and the upper broken tibia end was stretched. The stress concentration appeared in the unidirectional hinges connecting the moving platform and the adjustable/support rods. 

During the simulation, the average of the stresses on the section of the broken bone surface along the axis *o*_2_*z*_2_ is multiplied by the total area is defined as the tensile force simulation value $F_{z,2}^{s}$, as shown in Table 4.

On the outer surface of the broken bone, the maximum stress occurred at the Kirschner pinhole. Hence, the stresses on the surface were symmetrically distributed, and it was found that the maximum stress occurred at the broken bone end surface near the Kirschner wire pinhole, where points O and P were peak points close to the support and adjustable rods, respectively, as shown in Figure 10. 

**Figure 11:**
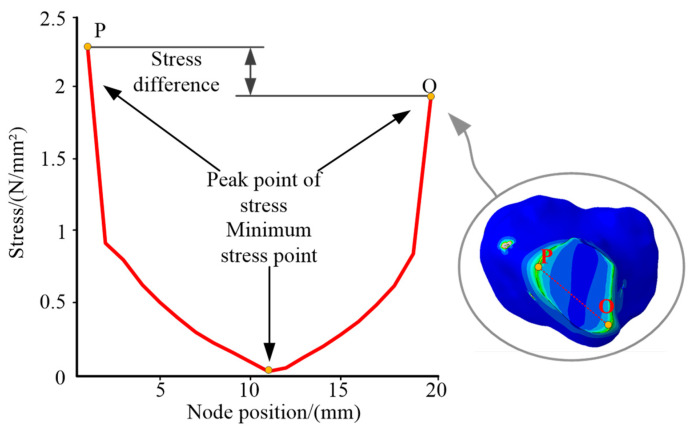
Stress distribution on the line OP.

The tensions $F_{z,2}^{s}$ affecting the broken bone segment lead to changes in the biological environment/skeleton morphology. Figure 11 shows a line OP connecting the points O and P, with the stresses distributed on the line OP. The stresses decrease from the peak point O and increase to another peak point P. The stress of point P is higher than that of point O. The difference indicated that the tibia was significantly affected by the tensions during the treatment process. The orthopedic treatment of the broken tibia could be completed with the help of the tensions.

### 3.4. Comparison of Theoretical, Simulation and Experimental Results

**Figure 12:**
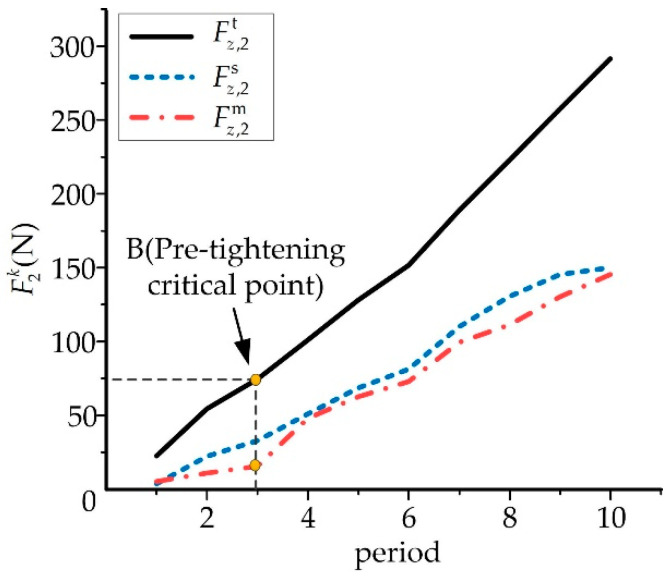
Comparison of the tensile force of the broken bone end.

In order to better compare the accuracy of the three methods, the corresponding results of the tensions are shown in Figure 12. Point B is the pre-tightening point of the unidirectional hinges during the experiment. Before pre-tightening, the hinges and rods were still in a loose state, which led to gaps between various parts. Hence, the measured tensions $F_{z,2}^{m}$ were less than the simulation tensions $F_{z,2}^{s}$. After point B, the adjustable forces reached the critical point of pre-tightening and the gaps among the components disappeared. The measured tensions $F_{z,2}^{m}$ increased faster. However, the curves of the tensions $F_{z,2}^{m}$ and $F_{z,2}^{s}$ had similar shapes. During the orthopedic experiments, various factors influenced the force transmission efficiency of the adjustable forces and led to the difference between the results $F_{z,2}^{m}$ and $F_{z,2}^{s}$. 

The theoretical/measured/simulation results increased synchronously according to the ratio of force conduction efficiency, since the theoretical data did not consider friction and there was no gap problem. Hence, there was no pre-tightening critical point in the theoretical analysis, in which the growth rate was twice that of the simulated value, and the growth rate was maintained at a stable rate.

Through comparing the tensions obtained by the three methods, it was found that the occurrence of the pre-tension critical point was related to the installation of the fixator, the adjustable forces and the synchronization of the physician. In the stage of orthosis, these problems will lead to unsatisfactory correction results and should be eliminated as much as possible in the correction periods to improve orthopedic treatments.

## 4. Discussion

The Ilizarov external fixator has attracted the attention of researchers due to its versatile configuration and good clinical results, and because it mainly changes the skeletal force line by fixing or limiting the movement of the limb to achieve the function of orthopedic limb bone, which has good clinical prospects for the treatment of knee osteoarthritis and limb orthopedics. Many researchers have achieved results in the study of external fixators. Thiart G, Herbert C, Sivarasu S, et al. studied the influence of different connecting rod structures on the stability of the Ilizarov external fixator [30]. However, the clinical application relies on physician experience, and the orthopedic features are still not clearly explained. This study analyzed the mechanical characteristics of the Ilizarov external fixator by combining theoretical analysis, clinical experiment and finite element analysis, which provide a reliable basis for the application of external fixators. The results are beneficial for the development of bone external fixation techniques.

First of all, based on the screw theory, the screw model of the Ilizarov external fixator was deduced innovatively and the geometric explanation of the fixator was given. Afterwards, in contrast with the study of Karunratanakul K, who used resin rodlets to conduct simulation experiments [10], orthopedic experiments were conducted using sheep tibia innovatively, and the theoretical tensions based on geometric explanation were compared with the experimental tensions obtained by orthopedic experiments on sheep tibia. It was found that there are interactions and constraints between the adjustable rod and the support rod due to the structural configuration of the external fixator. The transfer efficiency between the fixator and the broken bone end was calculated and analyzed. The analysis of the experimental results can provide a direction and theoretical basis for the innovative design of orthopedic parameters.

On this basis, a finite element model was established to accurately describe the stress and strain of the fixator and tibia during the process of orthopedic treatment. In contrast with the study of Donaldson F E, Pankaj P et al., who used pipe for simulation analysis [31], we used CT scans of tibia for modeling to obtain a more accurate model, and the model was able to respond to some phenomena that were difficult to obtain in the experiments. Similarly to the research results of Donaldson F E, the maximum stress appeared in the pinhole of Kirschner wire [32], and it can be seen from the results that the stress value of the pinhole near the adjustable rods was greater than that of the support rods. These results can provide a theoretical basis for the precise regulation of the external fixator and assist physicians in formulating special treatment plans. The finite element model shows some adaptability and can be applied to the optimization of the Ilizarov external fixator and other orthopedic simulations.

Ganesharajah, Ganadhiepan, Saeed, et al. proposed that callus healing was related to stress between broken bone ends [33]. Based on the established biomechanical model, biomechanical analysis and prediction can be performed to evaluate the degree of callus healing and bone alignment. However, it is difficult for in vitro animal experiments to simulate the muscle system. The influences of cartilage and ligaments on the orthopedic process have not been considered in theoretical calculations and experiments and simulations. In the future, the osteomuscular system of the biomechanical model will continue to be improved to obtain more accurate research results.

## Figures and Tables

**Table 1:** Classic parameters of Ilizarov external fixator.

Parameter	Value	Parameter	Value
Hole ring	2	Diameter of hole ring	190 mm
One-way hinge	4 pairs	Diameter of Kirschner wire	2.5 mm
Broken bone space	10 mm	Pulling speed	1 mm/d
Tibia varus angle	6.85°	Number of adjustments	10 times

**Table 2:** Finite element model parameters of fixator.

The Typical Sites	Number of Elements	Number of Nodes	Type of Mesh
Kirschner wire, steel wire holder	90,635	504,474	hexahedral mesh
tibia	67,973	127,614	tetrahedral mesh

**Table 3:** Average orthotic force in each treatment period.

Correction Period	*F*_1_/N	*F*_2_/N	$F_{z,2}^{m}/N$
1	0.56	8.56	5.26
2	12.18	20.78	11.03
3	16.89	30.31	15.58
4	27.28	40.03	48.11
5	35.81	53.04	59.75
6	43.17	63.83	72.83
7	58.65	77.15	93.52
8	70.51	91.92	111.37
9	90.77	99.39	130.25
10	99.62	117.54	145.39

**Table 4:** Tensions $F_{z,2}^{s}$ of the broken bone surface.

Period/Time	1	2	3	4	5	6	7	8	9	10
Tension/N	3.667	22.32	32.71	50.89	68.53	81.28	120.00	130.60	151.31	155.0

## Data Availability

Not applicable.
